# Comparison of Diagnostic Tests for *Onchocerca volvulus* in the Democratic Republic of Congo

**DOI:** 10.3390/pathogens9060435

**Published:** 2020-06-02

**Authors:** An Hotterbeekx, Jolien Perneel, Michel Mandro, Germain Abhafule, Joseph Nelson Siewe Fodjo, Alfred Dusabimana, Steven Abrams, Samir Kumar-Singh, Robert Colebunders

**Affiliations:** 1 Global Health Institute, University of Antwerp, 2000 Antwerp, Belgium; jolien.perneel@student.uantwerpen.be (J.P.); JosephNelson.SieweFodjo@uantwerpen.be (J.N.S.F.); Alfred.Dusabimana@uantwerpen.be (A.D.); Steven.Abrams@uantwerpen.be (S.A.); robert.colebunders@uantwerpen.be (R.C.); 2 Provincial Health Division Ituri, Ministry of Health, Bunia 185 DRC 57, Democratic Republic of Congo; Michel.MandroNdahura@student.uantwerpen.be; 3 Centre de Recherche en Maladies Tropicales, Rethy Box 143, Democratic Republic of Congo; abhafule@gmail.com; 4 Molecular Pathology Group, Laboratory of Cell Biology & Histology, Faculty of Medicine and Health Sciences, University of Antwerp, 2000 Antwerp, Belgium; Samir.Kumar-Singh@uantwerpen.be

**Keywords:** onchocerciasis, Onchocerca volvulus, antibodies, diagnosis, OV16 testing, microfilariae, epilepsy

## Abstract

Onchocerciasis is diagnosed by detecting microfilariae in skin snips or by detecting OV16 IgG4 antibodies in blood by either enzyme linked immunosorbent assay (ELISA) or a rapid diagnostic test (RDT). Here, we compare the sensitivity and specificity of these three tests in persons with epilepsy living in an onchocerciasis endemic region in the Democratic Republic of Congo. Skin snips and blood samples were collected from 285 individuals for onchocerciasis diagnosis. Three tests were performed: the OV16 RDT (SD Bioline) and the OV16 ELISA both on serum samples, and microscopic detection of microfilariae in skin snips. The sensitivity and specificity of each test was calculated with the combined other tests as a reference. Microfilariae were present in 105 (36.8%) individuals, with a median of 18.5 (6.5–72.0) microfilariae/skin snip. The OV16 RDT and OV16 ELISA were positive in, respectively, 112 (39.3%) and 143 (50.2%) individuals. The OV16 ELISA had the highest sensitivity among the three tests (83%), followed by the OV16 RDT (74.8%) and the skin snip (71.4%). The OV16 RDT had a higher specificity (98.6%) compared to the OV16 ELISA (84.8%). Our study confirms the need to develop more sensitive tests to ensure the accurate detection of ongoing transmission before stopping elimination efforts.

## 1. Introduction

Onchocerciasis is a disabling disease caused by infection with the filarial nematode *Onchocerca volvulus* and is linked to skin disease, blindness and epilepsy in remote areas of Africa and Latin America [1,2]. To reduce the onchocerciasis disease burden, the World Health Organization (WHO) and African Program for Onchocerciasis Control (APOC), now part of the Expanded Special Program for Elimination of Neglected Tropical Diseases (ESPEN), have started rigorous elimination campaigns with the community distribution of ivermectin (CDTI) [1,3,4,5]. When a country achieves the required interruption of onchocerciasis transmission to discontinue CDTI, many years of post-treatment surveillance still have to follow to ensure permanent elimination [6]. Current post-treatment surveillance guidelines to screen for ongoing transmission include the PCR pool screening of the blackfly vector and serological screening of children younger than 10 years old for the presence of OV16 antibodies [6,7,8].

OV16 IgG4 antibodies can be detected in dried blood spots or serum by an enzyme linked immunosorbent assay (ELISA), or using a rapid diagnostic test (RDT) [7,9]. The OV16 serology only detects exposure to the *O. volvulus* parasite and is therefore not informative about the current infection status. The sensitivity of the OV16 RDT is reported to be approximately 60–80%, whereas the specificity is estimated to be 99% [7,8,10]. This sensitivity is not high enough to detect the <0.1% seroprevalence proposed to stop onchocerciasis elimination efforts [6]. Moreover, it is not clear when seroconversion occurs: before or after the maturation of the adult worm or when the first microfilariae are produced [9,10]. Currently the OV16 RDT is used to determine transmission rates of onchocerciasis in epidemiological studies and is well accepted by the community [11]. Active onchocerciasis infection is diagnosed by the detection of microfilariae in skin snips usually taken from the left and right iliac crests. Although diagnosis by skin snip is highly specific and is considered to be the gold standard for onchocerciasis, it also has major disadvantages. For example, it is labor intensive and requires a well-trained lab technician, might be painful, is logistically challenging, time consuming and has a low sensitivity in areas with low microfilariae loads, such as after multiple rounds of CDTI [11,12,13].

In this study, we compare serological results obtained with the OV16 RDT and the OV16 ELISA with skin snips results from persons with epilepsy in an onchocerciasis-endemic region the Democratic Republic of Congo (DRC).

## 2. Materials and Methods

### 2.1. Study Setting and Design

Samples were collected during a cross-sectional onchocerciasis assessment in persons with epilepsy (PWE), as part of a clinical trial conducted in onchocerciasis-endemic villages in the Logo health zone, Ituri province, DRC [14,15]. In these villages (Draju, Kanga, Wala, Tedheja, and Ulyeko), ivermectin mass drug administration was never implemented. Previously, a high epilepsy prevalence (4.6%, 95% confidence interval: 3.6–5.8) had been documented in the area [16]. Among the 420 persons with epilepsy examined by Lenaerts et al., 67.6% met the diagnostic criteria of onchocerciasis associated epilepsy [17].

The study sites were essentially rural communities, with several fast-flowing rivers providing suitable breeding grounds for the blackfly vectors. The main economic activity of the residents was farming. All individuals who agreed to take part in the screening for the aforementioned clinical trial were eligible, even those who did not meet the inclusion criteria for the trial.

### 2.2. Study Participants and Sample Collection

Persons with epilepsy were asked to participate in the study and after informed consent was obtained, participants were interviewed and clinical data collected on a standardised questionnaire. Local health centres were used as recruitment grounds, where the research team established mobile clinics. Skin snips were taken from the left and the right iliac crests with a sterile corneoscleral punch (Holt, 2 mm) [18]. Blood samples were also obtained from each participants, immediately placed in a cold flask with ice, and transferred to a refrigerator upon returning to the laboratory on the same day. All procedures were performed following rigorous aseptic conditions.

### 2.3. Detection of O. volvulus in Skin Snips by Microscopy

Each skin snip was transferred to a single well of a microtitre plate and a few drops of saline was added. Biopsies were incubated for 24 h to allow the microfilariae to emerge from the tissue and a count was performed under a microscope. A person was considered to be infected when *O. volvulus* microfilariae were present in his skin.

### 2.4. Detection of Antibodies against O. volvulus

**Figure 1:**
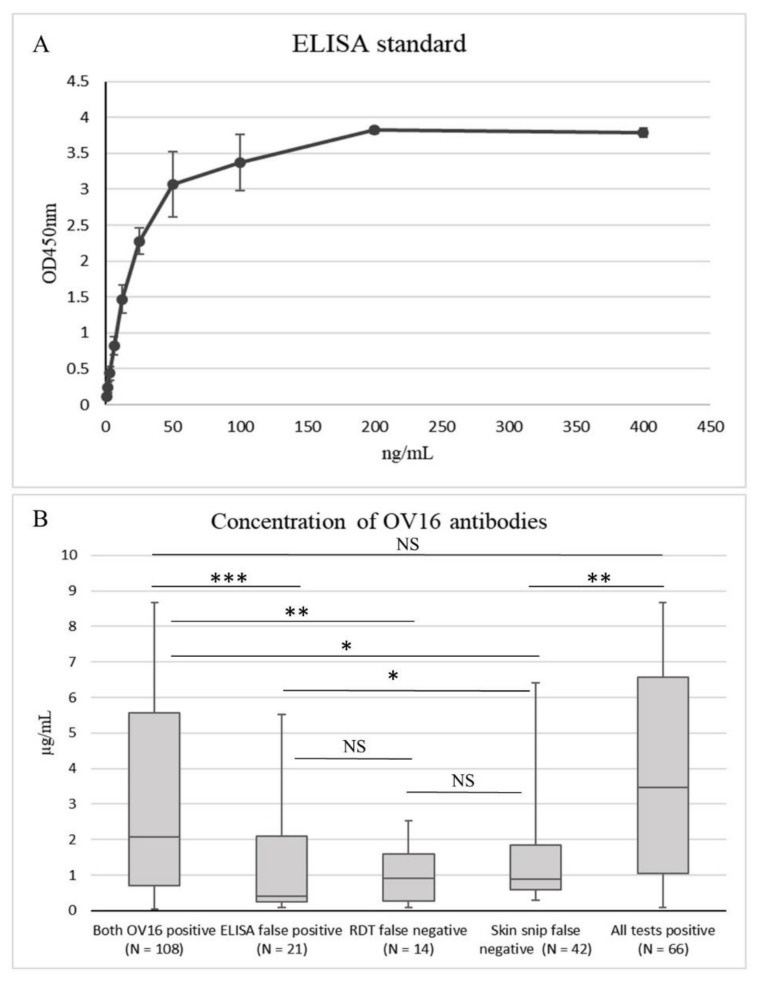
(**A**) Concentration-dependent absorbance values of the OV16 ELISA standards. The lowest concentration tested was 0.78125 ng/mL. (**B**) Concentration of OV16 antibodies. Both OV16 positive: all samples with 2 positive OV16 tests and positive or negative skin snip results; ELISA false positive: negative for OV16 RDT and skin snip; RDT false negative: positive for OV16 ELISA and skin snip; skin snip false negative: negative result for skin snip but positive for both OV16 tests. NS: not significant; * *p* < 0.05; ** *p* < 0.01;*** *p* < 0.001. Wilcoxon Rank Sum test.

The OV16 rapid diagnostic test (OV16 RDT) was performed by trained health care workers, according to the manufacturer protocol (SD Bioline Onchocerciasis IgG4 rapid test, Abbott Standard Diagnostics, Inc., Yongin, Republic of Korea). This test qualitatively detects IgG4 antibodies against the OV16 antigen and the results are immediately available after the recommended 20 minute incubation. Additionally, serum was screened for OV16 IgG4 antibodies by a non-commercial enzyme-linked immunosorbent assay (ELISA) based on horseradish peroxidase-based product detection, as described earlier [19]. Commercially available recombinant OV16 IgG4 (Bio-Rad AbD Serotec, Puchheim, Germany) was used to make a standard curve in every assay (Figure 1A), which was used to calculate the OV16 IgG4 antibody concentration in the serum samples [19]. Briefly, 96-well plates (Thermofisher Scientific, Merelbeke, Belgium) were coated with OV16 antigen (CUSABIO, Cambridge, UK) overnight. Serum samples were diluted 1:200 in each assay. HRP-conjugated mouse anti-human IgG4 Fc antibodies (HP6025, Abcam, Cambridge, UK) were used as a detection antibody, diluted 1:10000 and incubated for 1h. The outcome of the OV16 ELISA was compared to skin snip positivity, and to the OV16 RDT.

### 2.5. Data Processing and Statistical Analysis

Data processing and statistical analysis were performed in R version 3.6.3 and Microsoft Excel 2010. Only individuals with test results for all three diagnostic tests were included. Sensitivity and specificity were calculated for each test, and, to do so, true and false results were determined by comparing the result of a given test with the combined results of the other two with the following considerations: a positive skin snip is always a true positive even when serology is negative for both OV16 tests. In this case, the serology is considered false negative. A negative skin snip but two positive OV16 tests (RDT and ELISA) was considered as an active *O. volvulus* infection with low microfilarial load, in the absence of ivermectin treatment. In case of discrepancy between the RDT and ELISA OV16 tests, the skin snip result is used as a golden standard to determine which result is true and which is false. Median concentrations of OV16 antibodies were compared for different groups using the Wilcoxon Rank Sum test. P-values below 0.05 were considered significant. The inter- and intraplate coefficient of variation was calculated as a quality control for the OV16 ELISA.

### 2.6. Ethical Considerations and Informed Consent

The study was approved by the Ethics Committee of the School of Health of the University of Kinshasa, DRC, and the University of Antwerp, Antwerp, Belgium. All eligible candidates provided a written informed consent before enrolment into the study.

## 3. Results

### 3.1. Demographic and Clinical Information of the Study Participants

In total, 285 persons with epilepsy (PWE) were included, with a median age of 21 years old (IQR: 15–30) (Table 1). Of those, 147 (51.6%) were male. Only six (2%) individuals of the total study population reported a history of ivermectin use at the time of the study. Clinical examination revealed that the majority of people (85.3%) had normal skin, although 105 (36.8%) reported itching. Forty-two (14.7%) individuals were reported with skin abnormalities, 8 (19.0%) presented with papular skin, 10 (23.8%) had leopard skin, 12 (28.6%) had lizard skin and 18 (42.8%) did not specify the abnormality. Palpable onchocercal nodules were observed in 17 (5.8%) of all PWE, whereas 105 (36.8%) had microfilariae in their skin snips, with a median infection load of 18.5 (IQR: 6.5–72.0) microfilariae/skin snip. Notably, only 44 persons with a positive skin snip also reported itching, whereas 60 persons with a positive skin snip did not report itching and 61 persons who reported itching had a negative skin snip. There was no significant correlation between both (*p* = 0.1557). Serology revealed 112 (39.3%) positive OV16 RDT tests and 143 (50.2%) positive OV16 ELISA results.

### 3.2. Comparison of the Three Diagnostic Tests

The OV16 ELISA had the highest sensitivity among the three tests (83%), followed by the OV16 RDT (74.8%) and the skin snip (71.4%) (Table 2). The skin snip had the highest specificity (100%) followed closely by the OV16 RDT (98.6%) and ELISA (84.8%). Twenty-three (8.1%) participants had not (yet) developed an antibody response despite the presence of microfilariae in their skin snips, whereas 42 (14.7%) did not have microfilariae in their skin snips but developed an antibody response detected by both RDT and ELISA. There was no significant difference in the median skin mf load between the persons with a positive OV16 serology (15.0 mf/skin snip, IQR: 6.5–57.4) or a negative OV16 serology (52.5 mf/skin snip, IQR: 9.0–115.3; *p* = 0.054). Similarly, there was also no difference in median age between the persons with positive OV16 serology but negative skin snip (24.5 years, IQR: 18.5–30.75) and the persons with a positive skin snip and negative OV16 serology (21 years, IQR: 15.5–31.0; *p* = 0.438). Overall, 147 (51.6%) PWE were infected with *O. volvulus* (true positive + false negative results).

### 3.3. OV16 Antibody Concentration

There was a significant difference in the median OV16 antibody concentration, depending on how many tests were positive (*p* < 0.001, Figure 1B). The median OV16 antibody concentrations were the highest in the individuals with a positive result for all three tests (3.457 µg/mL) and with a positive result for both OV16 tests (2.084 µg/mL) (Figure 1B). The lowest median OV16 antibody concentration (0.402 µg/mL) was found in the individuals with only a positive result for the OV16 ELISA test, which is considered a false positive test result. However, the median OV16 antibody concentration of the false positive ELISA test was not significantly different from those with a false negative OV16 RDT and a positive skin snip result (median = 0.911; *p* = 0.828) and those with a false negative skin snip result and a positive OV16 RDT result (median = 0.895; *p* = 0.104, Figure 1B). Figure 1B shows the concentration-dependent absorbance values of the positive control used in the OV16 ELISA assay. The lowest concentration tested was 0.78125 ng/mL. The interplate coefficient of variation was 18.5% and the intraplate coefficient of variation was 4.2%.

## 4. Discussion

In our study, the OV16 ELISA test had a slightly higher sensitivity (83%) compared to the OV16 RDT (74.8%), and the RDT had a slightly higher specificity (98.6%) compared to the ELISA test (84%). The performance of the serology tests was in the same range as published earlier [7,8,10]. Only 17 (11.6%) of the infected individuals, who were never exposed to ivermectin, had palpable onchocercal nodules and 42 (14.7%) showed abnormal skin. A potential explanation could be that the population is relatively young and is therefore more likely to have a relatively recent infection and not yet developed prominent skin abnormalities. Indeed, both the number of nodules and skin abnormalities are increasing with age [20,21,22]. However, it is possible that some nodules have been missed because it is not easy to detect nodules that are small or located in areas where they are uncommon to occur, such as deep tissues.

Forty-two individuals had negative skin snip results but a positive serology. However, the OV16 antibody concentration observed in these individuals was lower compared to individuals who had positive skin snip results. These individuals either had very low microfilarial densities, below the detection limit, or had a recent infection while already having antibody production but no microfilariae production. It has been reported that antibodies become detectable 1–2 years before microfilariae appeared in the skin [23]. On the other hand, in our study, 23 individuals had a positive skin snip but had not (yet) developed a detectable antibody response against the OV16 antigen. The mechanism behind the generation of antibodies to specific parasite compounds is dependent on the parasite as well as the immune response, and, sometimes, the antibodies appear after the microfilariae [23]. We do not know whether some of these 23 individuals might have developed OV16 IgG4 antibodies later, because no follow up study was done. However, the most likely explanation of the false negative OV16 result is that the test is not sensitive enough. Individuals with a false negative OV16 RDT had low OV16 antibody concentrations, similar to those with a false negative skin snip result, because the limit of detection is lower in the ELISA test. Individuals with a false positive ELISA test had the lowest antibody concentrations, raising the question of whether these are truly false positive results or the actual sensitivity of this test might be higher than estimated based on the other tests. A test combining different onchocerciasis biomarkers will be needed to increase the sensitivity to detect ongoing transmission [24].

A limitation of this study is that skin snips were not tested for the presence of *O. volvulus* DNA by quantitative PCR, the golden standard for the detection of an *O. volvulus* infection. Indeed, the sensitivity of skin snip microscopy was found to be 62.3% compared to a quantitative *O. volvulus* PCR test on skin snips and 40.4% in skins with <2 microfilariae/skin snip [25].

In conclusion, the skin snip microscopy and OV16 RDT show equal performance to detect *O. volvulus* infection in a population where ivermectin has never been distributed. The OV16 ELISA test has a slightly higher sensitivity but lower specificity compared to the other two tests. More research is needed to determine the optimal tools for onchocerciasis diagnosis, especially in regions with ongoing elimination programs and low community microfilariae loads, to ensure the accurate detection of ongoing transmission before deciding to stop elimination efforts.

## Figures and Tables

**Table 1:** Demographic and clinical information of the study population.

Characteristics	Persons with Epilepsy (n = 285)
Age: median (IQR)	21 (15–30)
Male: n (%)	147 (51.6%)
Weight (kg): median (IQR)	45.8 (35–51)
Height (cm): median (IQR)	152 (143–160)
Received ivermectin: n, (%)	6 (2%)
Itching ^1^: n (%)	105 (36.8%)
Abnormal skin ^#^: n (%)	42 (14.7%)
Papular skin: n (%)	8 (19.0%)
Leopard skin: n (%)	10 (23.8%)
Lizard skin: n (%)	12 (28.6%)
Unspecified: n (%)	18 (42.8%)
Onchocerca nodules ^2^: n (%)	17 (5.9%)
*O. volvulus* microfilariae present in skin snip: n (%)	105 (36.8%)
Microfilariae load: median (IQR)	18.5 (6.5–72.0)
Positive OV16 ELISA: n (%)	143 (50.2%)
Concentration OV16 antibodies (µg/mL): median (IQR)	1.66 (0.47–4.46)
Positive OV16 RDT: n (%)	112 (39.3%)
*O. volvulus* infection *: n (%)	147 (51.6%)

^1^ 8 missing data; ^2^ 7 missing data; ^#^ Some individuals reported multiple skin abnormalities; IQR: interquartile range; RDT: Rapid diagnostic test; * *O. volvulus* infection was determined by a positive skin snip and/or a positive result for both OV16 tests.

**Table 2:** Comparison of the three diagnostic tests. A positive skin snip is always considered a true positive, and a negative skin snip with two positive OV16 tests is considered a false negative. The skin snip test result is followed in case of discrepancy between the OV16 tests.

	Skin Snip	OV16 ELISA	OV16 RDT	Total
All negative	-	-	-	115
All OV16 false negative	+	-	-	23
ELISA false positive	-	+	-	21
RDT false negative	+	+	-	14
RDT false positive	-	-	+	2
ELISA false negative	+	-	+	2
Skin snip false negative	-	+	+	42
All positive	+	+	+	66
True Negative	138	117	136	NA
False Negative	42	25	37	NA
True Positive	105	122	110	NA
False Positive	0	21	2	NA
Sensitivity	71.4%	83%	74.8%	NA
Specificity	100%	84.8%	98.6%	NA

-: Negative test result; +: Positive test result; NA: not available.
